# Pregnancy and Lactation in a 67-Year-Old Elderly Gravida following Donor Oocyte *In Vitro* Fertilization

**DOI:** 10.1155/2020/9801565

**Published:** 2020-09-12

**Authors:** Leila Magistrado, Mary C. Tolcher, Anju Suhag, Sonal Zambare, Kjersti M. Aagaard

**Affiliations:** ^1^Department of Obstetrics & Gynecology, Division of Maternal-Fetal Medicine, Baylor College of Medicine, Houston, TX, USA; ^2^Department of Anesthesiology, Division of Obstetric Anesthesia, Baylor College of Medicine, Houston, TX, USA; ^3^Department of Molecular & Human Genetics, Baylor College of Medicine, Houston, TX, USA; ^4^Department of Molecular & Cell Biology, Baylor College of Medicine, Houston, TX, USA

## Abstract

There is limited data on the anticipated perinatal course among gravidae in their sixth and seventh decades. Our objective was to describe the relatively uncomplicated prenatal, intrapartum, and postpartum course of a 67-year-old essential primigravida. Briefly, our patient conceived a singleton pregnancy via IVF with donor oocytes, then presented at 13 6/7 weeks of gestation to initiate prenatal care. Her medical history was significant for chronic hypertension, hyperlipidemia, and obesity. Her cardiac function was monitored throughout pregnancy, and she delivered at 36 1/7 weeks by cesarean for a decline in left ventricular function with mitral regurgitation. Her intrapartum and postpartum course was uncomplicated, and she was able to successfully breastfeed for six months and resume prepregnancy activity. For comparison, we analyzed deliveries among gravidae > 45 years of age from our institutional obstetrical database (2011-2018). This case represents the eldest gravidae identified in the literature and illustrates the potential for a relatively uncomplicated perinatal course with successful lactation. This case may enable other providers to counsel elderly patients on anticipated outcomes inclusive of ability to breastfeed.

## 1. Introduction

Pregnancy among gravidae past the sixth decade has become possible with assisted reproductive technology (ART) using oocyte donation. With the development of ART and elective postponement of parenting, the occurrence and prevalence of pregnancy at advanced maternal age have increased. According to the Centers for Disease Control and Prevention (CDC), rates of pregnancy for women in the United States aged 30 and above have increased over the last 20 years, with women over aged 40 having the largest percent increase (70% in 20 years) [1]. “Elderly” gravidae (defined as ≥40 years of age) have been observed to bear increased risk of maternal and fetal morbidity, including preeclampsia, gestational diabetes, fetal anomalies, and preterm delivery [2]. However, the occurrence of many of these pregnancy disorders further varies by preexisting comorbidities, such as chronic hypertension, which renders both personalized counseling and tailored management of elderly gravidae challenging.

These challenges are further confounded by increased availability and utilization of *in vitro* fertilization (IVF) globally. The first successful pregnancy by IVF was reported in 1976 [3]. Since then, the rate of pregnancies achieved worldwide by ART including IVF have increased and currently account for 1.7% of live births in the United States (2015 estimates) [4].

While there has been much discussion regarding establishing an upper age limit for ART given the increased risk of complications to both the mother and the infant, any established limitations must be guided by an evidence base and balance any individuals' personalized risk and reproductive choice [5]. In the United States, between 1997 and 1999, 539 births were reported among mothers over age 50, with 194 being over 55 [5]. According to the Guinness World Records, the previously oldest verified mother was aged 66 years (http://www.guinnessworldrecords.com/world-records/oldest-person-to-give-birth). Here, we present the relatively uncomplicated course of pregnancy and lactation in the oldest verified mother at age 67, with delivery at 68 years of age. This essential primigravida presented for care following donor oocyte IVF elsewhere.

## 2. Case Presentation

Informed consent from the patient was obtained, and she provided explicit written consent. Specifically, she confirmed that she was the person depicted in the article tentatively entitled “Pregnancy and Lactation in a 67-year-old Elderly Gravida following Donor Oocyte *In Vitro* Fertilization.” She granted written informed consent without restriction for publication of her material, excluding photographs. She understands that while her name would not be published, complete anonymity could not be guaranteed. She was offered the opportunity to read the manuscript in which she was included. Since this did not constitute human subjects research, IRB approval for the case report was deferred by the Baylor College of Medicine IRB. The database query was conducted under IRB approval (Baylor College of Medicine IRB H-43835).

Our patient is a 67-year-old G5P0040 with a history of four first trimester pregnancy losses. The pregnancy was conceived via IVF with oocyte donation (a presumptively healthy 22-year-old female) and the 74-year-old husband's sperm in West Africa; no records nor details regarding the initial evaluation or IVF procedures were available, and unconfirmed details are thus not reported. The patient's medical history was significant for chronic hypertension, hyperlipidemia previously maintained on simvastatin, and class I obesity (body mass index with a prepregnancy value of 32 kg/m^2^). Her chronic hypertension was currently well controlled off of medications, although one year prior to initiating IVF she was on an angiotensin-converting enzyme inhibitor (lisinopril).

At her first clinic visit to our obstetrics high-risk clinic at 13 6/7 weeks of gestation, baseline laboratory studies and vital signs were obtained (Table 1). Her blood pressure was well controlled without medication. Based on her age and history of chronic hypertension, she was initiated on aspirin 81 mg daily due to her increased risk of preeclampsia [6, 7]. However, there are no consensus guidelines regarding management of comorbidities at the extremes of maternal age. Thus, during our weekly multidisciplinary conference, group consensus among our team of maternal-fetal medicine (MFM) specialists and obstetrical anesthesiologists was reached and included baseline assessment of cardiac function with echocardiogram and screening for renal and hepatic dysfunction as recommended by the American College of Obstetricians and Gynecologists (ACOG) [6, 7]. In addition, our team felt that single time point examination at such a marked maternal age (67 years) may fail to detect any accelerated disease process and recommended serial examinations including echocardiograms, comprehensive metabolic profile, brain natriuretic peptide, and 24-hour urine protein collection and analysis every six to 12 weeks. Subsequent patient visits during pregnancy were uneventful. Baseline echocardiography was initially delayed due to challenges in obtaining coverage for obstetrical indications under Medicare coverage, which she was mandated to due to her age; it was obtained in the early 3^rd^ trimester (Table 1). Of note, the American Heart Association (AHA) reports that the incidence of CVD among men and women in the U.S. approximates 40% from 40 to 59 years of age, 75% from 60 to 79 years of age, and 86% among those above the age of 80 [8].

At approximately 31 weeks of gestation, she presented to her ultrasound appointment with persistent cough not relieved by cough suppressants for several weeks and four days of lower extremity swelling. She was directly admitted for further evaluation for heart failure and/or superimposed preeclampsia. Work-up included labs (brain natriuretic peptide (BNP), thyroid-stimulating hormone (TSH), free T4, and preeclampsia labs), lower extremity Doppler, chest X-ray, electrocardiogram, and echocardiogram. Labs and lower extremity Doppler compression studies were negative. Chest X-ray showed moderate cardiomegaly and mild central pulmonary venous congestion. Electrocardiogram showed sinus rhythm. Echocardiogram showed normal left ventricular systolic function with ejection fraction of 70%. The patient was deemed stable and discharged on day 2 of hospitalization. She continued prenatal care with her primary MFM provider thereafter.

At 35 6/7 weeks of gestation, a new-onset II/VI holosystolic murmur, heard loudest over the mitral valve with radiation to the lateral aspect of the apex of the heart and thus concerning for mild to moderate mitral regurgitation, was noted on her weekly cardiac auscultation. These cardiac findings were accompanied by bilateral 2+ pitting edema, which in turn raised concern for superimposed preeclampsia (Table 1). Repeat echocardiogram was significant for decreased left ventricular ejection fraction from 70% to 60-64% with new-onset trace mitral regurgitation (Table 1). The shared patient-physician decision to proceed with primary lower transverse cesarean delivery was made under full and informed consent. An uneventful single subarachnoid injection (spinal) was performed in the operating room, and preservative-free intrathecal morphine was used for postoperative analgesia. Fluid management during the cesarean was kept conservative, and the patient was monitored closely for postoperative respiratory depression and other rare postanesthetic complications such as nerve damage, infection, and/or postdural puncture headache for the next 24 hours. Given only trace mitral regurgitation on her predelivery echocardiogram and stable clinical exam finding, a repeat echo exam postpartum was not conducted. She had just reached 68 years of age at the time of delivery.

Cesarean delivery was uneventful with successful delivery of a female neonate, Apgar scores 7 and 9, weight of 2520 grams. Her postpartum course was uneventful with improvement of lower extremity edema and normotensive blood pressures not requiring additional medical management. She was discharged on postpartum day three and was successfully breastfeeding after lactation consultation. At six weeks postpartum, her Pfannenstiel incision site was well healed, she continued to exclusively breastfeed with mother's own milk, and she showed no signs or symptoms of postpartum depression or cardiac compromise. Her blood pressure remained well controlled off of medications, and she planned for ongoing follow-up with internal medicine for her chronic medical conditions.

Inspired by this case, under IRB approval (Baylor College of Medicine IRB H-43835), we conducted a query of our perinatal database to characterize maternal risks among gravidae > 45 years of age. Of 30,131 deliveries in our database (2011-2018), 6319 were advanced maternal age (AMA, 21%); *n* = 83 were ≥45 years, and *n* = 6236 were 35-44 years old. Chronic hypertension (*p* = 0.031), nulliparity (*p* = 0.040; SPSS, IBM Corporation), *in vitro* fertilization (IVF) resultant pregnancy (*p* < 0.001), and multiples (*p* < 0.001) were significantly more common among gravidae ≥ 45 years. In terms of pregnancy outcomes, preterm delivery ≥ 34 and <37 weeks was more common in the ≥45 years of age group (OR 2.41, 95% CI 1.48-3.92), but not delivery < 34 weeks (OR 1.81, 95% CI 0.78-4.20). The increased risk of preterm delivery < 37 weeks persisted after adjustment for ethnicity/race, IVF pregnancy, and multiple gestations (aOR 1.97, 95% CI 1.12-3.46). There was no difference in preeclampsia or cesarean delivery (*p* > 0.05). NICU admission was higher (*p* = 0.005), and mean birth weight was lower in the ≥45 group (*p* < 0.001) but to a clinically insignificant degree when our analysis was limited to singletons. These findings are consistent with our current case presented here. Overall, the risks of women who are ≥45 compared to 35-44 are not substantially elevated after controlling for potential confounders.

## 3. Discussion

This case brings forth several issues and topics fundamental to the care of elderly gravidae. The topics of advanced maternal age, advanced paternal age, anesthesia considerations, breastfeeding, ethics, and neonatal outcome are discussed further below.

### 3.1. Perinatal Risks in Gravidae of Markedly Advanced Maternal Age and Impact of Paternal Age

Advanced maternal age (maternal age at delivery of 35 or more) is associated with increased maternal and fetal risks including pregnancy loss, fetal anomalies, hypertensive disorders, gestational diabetes, cesarean delivery, and stillbirth, particularly in the setting of a first pregnancy at advanced age [5–10]. Studies examining women of extremely advanced maternal age (≥45 years) confirm known risks of advanced maternal age and show additional increased risks of placenta previa, postpartum hemorrhage, and adverse neonatal outcomes [11, 12]. Evidence for women ≥ 50 years is limited, but in one series of 45 live births among women aged 50-63 who received donated oocytes, 35% experienced hypertensive disorders of pregnancy (60% of those > 55 years old), 20% had gestational diabetes, and 78% underwent cesarean delivery [13]. Nulliparous women ≥ 50 years have a more than 2-fold increased risk of undergoing an intrapartum cesarean delivery [14]. With regard to positive outcomes, one study demonstrates a positive association between older maternal age and longevity. A nested case-control study using Long Life Family Study (LLFS) data showed that women who had their last child beyond the age of 33 years had twice the odds of survival to the top 5th percentile of survival of their birth cohorts compared to women who had their last child by age 29 [15]. In our case report, both parents are alive raising their daughter in a stable family household. Despite concerns for superimposed preeclampsia, at her postpartum visit, this 68-year-old elderly gravida continued to be well controlled without need of initiation of any antihypertensive medication.

Advanced paternal age risks are poorly understood or may have gradual effects [16]. However, advancing paternal age has been associated with epigenetic changes and increased risk of genetic diseases in offspring including single-gene mutations, autism, and schizophrenia; the precise rate difference of these genetic disorders as a product of paternal age is unknown [16, 26]. In our patient, genetic counseling and prenatal testing were offered for her partner's advanced paternal age, and findings of soft markers of aneuploidy on comprehensive fetal anatomy survey at 19 1/7 weeks of gestation were inclusive of a possible ventricular septal defect and borderline pyelectasis. Prenatal diagnosis via amniocentesis for fetal aneuploidy, chromosomal microarray analysis (CMA), noninvasive prenatal testing (NIPT), comprehensive second-trimester fetal anatomy survey via ultrasound, fetal echocardiogram, carrier screen test, and parental karyotype were offered to our patient. She elected to proceed with noninvasive prenatal testing (NIPT) and fetal echocardiogram. A fetal echocardiogram demonstrated biventricular hypertrophy but no evidence of septal defect. NIPT testing demonstrated an expected fetal fraction of chromosomes 21, 18, and 13 consistent with a female fetus with no apparent abnormality in the sex chromosomes. She declined diagnostic genetic testing.

### 3.2. Intrapartum Management and Mode of Delivery

In a shared decision model with the patient, we undertook mode of delivery counseling with the aim of understanding the patient's goals for her delivery, including the risks and benefits of attempted vaginal versus elective cesarean delivery in the context of advanced maternal age. In a population-based study comparing all singleton term deliveries, multiple logistic regression analysis suggested that nulliparity (OR = 3.8, 95% CI 3.3–4.3) and maternal age > 35 years (OR = 3.0, 95% CI 2.6-3.6) were independent risk factors for cesarean delivery due to failure to progress [17]. We apprised her of these risks but stated that we had no evidence base in her situation to guide our decision-making. She elected for a cesarean delivery.

While our patient had an uncomplicated anesthetic course, there are several general considerations in providing optimal care for an elderly patient. There are limited data on the anesthetic management in the elderly parturient, particularly in this age group. The performance of neuraxial anesthesia in the aging population alters the absorption, distribution, and clearance of the local anesthetics. This patient population may also present with technical difficulties while placing the neuraxial block due to age-related changes in the spine. The level of sensory blockade and the incidence of hypotension may be higher in the elderly with the same amount of intrathecal drug as compared to a younger patient [18]. The use of neuraxial morphine has been associated with an increased incidence of delayed respiratory depression after up to 24 hours in patients with advanced age [19]. Therefore, monitoring as recommended by the guidelines provided by the American Society of Anesthesiologists (ASA) is crucial. Given the known increased risks of postpartum hemorrhage and preeclampsia at this age, the obstetric anesthesiology team should take into consideration the effects of thrombocytopenia, hemodynamic changes, and airway changes as well as secondary effects of magnesium sulfate in preeclamptic patients. All of these effects can be exaggerated in the elderly parturient [20]. Additionally, patients in this age group should also be monitored for postoperative cognitive dysfunction.

### 3.3. Postpartum Lactation

With aging, the loss of breast epithelial tissue is defined as age-related lobular involution. Lobular involution begins in perimenopause and accelerates during menopause and is characterized as a decrease in the size and complexity of the ductal tree and of the terminal ductal lobular units [21]. This phenomenon would suggest a decrease in ability to breastfeed. However, there is limited data on the effect of lobular involution in advanced maternal age and breastfeeding. Breast milk production is regulated by the hormones prolactin and oxytocin; prolactin stimulates the growth and development of mammary tissue for the production of milk [22] while oxytocin is responsible for milk secretion. Our patient was able breastfeed her infant, contrary to reports that primiparous women of advanced maternal age are unable to breastfeed [23]. Her ability to breastfeed demonstrates an intact hypothalamic-pituitary axis required to produce prolactin and oxytocin which offered known benefits of breastfeeding including bonding and maternal and neonatal health benefits.

### 3.4. Ethics of Assisted Reproductive Medicine in Elderly Gravidae

The American Society for Reproductive Medicine (ASRM) states embryo transfer should be strongly discouraged or denied to women of advanced reproductive age with underlying conditions that increase or exacerbate obstetrical risks [24]. There are certainly a number of concerns which underlie this recommendation. For example, due to concerns related to the high-risk nature of pregnancy, as well as parental longevity, treatment of women over the age of 55 is generally discouraged, particularly in women with underlying medical disorders including hypertension or diabetes [25]. The U.S. maternal mortality rate for women aged 40 and over (81.9 per 100,000 live births) is nearly 8 times that for women under age 25 (10.6 per 100,000 live births; https://www.cdc.gov/nchs/data/nvsr/nvsr69/nvsr69-02-508.pdf). Parental longevity refers to a potential lack of psychosocial support of the child into adulthood. With a life expectancy of approximately 80 years for women in the United States (and approximately 72 for men), our patient would not be expected to live into the teenage years of her child. Parental loss is a stressful life event, particularly for children; however, it is difficult to absolutely determine an age at which the expected death of a parent is acceptable. It is clearer, though, that children would not be expected to live independently until at least the age of 18. Of note, the life expectancy of women in West Africa is 57 years.

We concur that discussions around oocyte and embryo donation in women of advanced age involve a balance of patient autonomy (individual rights to make reproductive choices and access reproductive services) and nonmaleficence (utilizing medical advances to overcome natural limitations that may result in increased maternal risks and concerns about childhood welfare and early parental loss). Whether paternal age should be considered and to what extent is unclear. Moreover, due to small numbers, there are limited data on the safety and pregnancy outcomes of women in their 50s or older, so counseling patients on what to expect is challenging. It is for these reasons that we feel our case report herein is of intrinsic and important clinical value. Without evidence to guide counseling and shared patient-provider decision-making, we are reliant on largely hypothetical risks and expert opinion. Our case brings to light the notion that there may be instances when there is no immediate risk of mortality to the elderly gravidae, and good outcomes may be experienced. However, we are mindful that higher risk of mortality may occur in a resource-limited setting or in instances when a patient presents late to care with advanced cardiac disease and worse clinical symptoms. While no single case report or case series should ever replace best clinical practice recommendations and good clinical judgment, there will be instances and cases (such as ours herein) where patients have undergone donor oocyte IVF elsewhere and will require our best clinical care through their elected pregnancy. It is our hope that this report will provide some framework for clinicians and their patients to engage in structure conversation.

### 3.5. Neonatal Outcome

On the two-month well-child examination, their infant had met their developmental milestones and no abnormalities were noted on physical exam, newborn screening, or postnatal echocardiogram. The following findings were noted on her two-month well-child exam, “baby smiles spontaneously, regards her own hand, grasps rattle when touched, follows object with eyes 180 degrees, vocalizes with ‘oohs, aahs,' squeals, turns to rattling sound, lifts head up to 90 degrees, keeps head steady when sitting, bears weight on legs, and lifts chest up when prone.” On physical exam, noted to be well-developed and well-nourished and newborn screening normal. With regard to concern for fetal ventricular septal defect on prenatal ultrasound, postnatal echocardiogram at Texas Children's Hospital was normal with no further need for postnatal follow-up. At the last follow-up contact nearly three years postdelivery, our patient reported having had successfully breastfed her infant to 18 months of age. Their child was verbal during the follow-up visit, and her parents relayed that all developmental milestones had been reached. Both parents remained alive and well, and each communicated their ongoing delight to be parenting an active and curious preschool child at their life stage.

In conclusion, there are limited data on pregnancy outcomes among women in the fifth and sixth decades of life. With trends in delaying childrearing and advancements in reproductive technologies, practitioners are likely to encounter more patients like ours. Ideally, preconception medical evaluation should be performed and medical conditions optimized prior to pregnancy, particularly in women over age 45. Patient counseling should include issues of medical and obstetrical complications and psychological and ethical considerations and should balance patient autonomy and practitioner nonmaleficence when risks are high.

## Figures and Tables

**Table 1:** Vital signs, echocardiogram findings, and significant lab values by gestational age.

Gestational age (weeks)	12 3/7	13 5/7	19 6/7	28 5/7	30 5/7	35 6/7	36 1/7	6 weeks postpartum
Vital signs								
Blood pressure (mmHg)	137/83	121/79	121/73	122/76	129/71	136/75	142/78	143/84
Pulse	93	76	73	81	76	69	67	63
Respiratory rate	20	17	16	16	18	18	16	16
SaO_2_ %	97	n.d.	n.d.	98	97	n.d.	96	n.d.
Weight (kg)	n.d.	73.0	74.8	n.d.	n.d.	n.d.	79.8	66.7
Labs								
Hemoglobin (g/dL)	11.3	n.d.	n.d.	n.d.	9.8	n.d.	11.0	n.d.
Platelet count (10^3/^)	232	n.d.	n.d.	n.d.	180	n.d.	189	n.d.
Urine protein (mg/24 hours)	n.d.	208	n.d.	202	n.d.	n.d.	n.d.	n.d.
Total protein/Cr (ratio)	n.d.	n.d.	n.d.	n.d.	0.12	n.d.	0.18	n.d.
BUN/Cr (mg/dL)	5/0.5	6/0.41	n.d.	3/0.48	4/0.43	5/0.5	3/0.5	n.d.
ALT/AST (U/L)	n.d.	19/21	n.d.	16/22	11/18	10/17	11/16	n.d.
BNP (pg/mL)	n.d.	n.d.	25	37	22	74	74	n.d.
Echocardiogram (TTE)								
Gestational age (weeks)	30 6/7				35 6/7			
TTE findings								
LVEF (%)	70%				60-64%			
Ventricular size	Normal				Normal			
Wall motion abnormality	No regional wall abnormality				Impaired relaxation			
Cardiac output (L/min)	10 L/min				n.d.			
Mitral valve	Structurally normal				Mild mitral valve thickening, trace mitral regurgitation			
Tricuspid valve	Structurally normal				Structurally normal			
Aortic valve	Structurally normal				Structurally normal			
Pulmonic valve	Structurally normal				Structurally normal			
Tricuspid regurgitation	Trace				n.d.			
RV systolic pressure (mmHg)	35-40 mmHg				n.d.			
RA pressure (mmHg)	0-5 mmHg				n.d.			

n.d. = not done (either not clinically indicated or unable to be performed).
